# Forced Sedentariness and Sports Activity as Factors Differentiating Anthropometric Characteristics, Indices, and Body Composition in People with Disabilities

**DOI:** 10.3390/biology11060906

**Published:** 2022-06-13

**Authors:** Anna Zwierzchowska, Barbara Rosołek, Marcin Sikora, Diana Celebańska

**Affiliations:** 1 Institute of Sport Sciences, Department of Biomechanics, The Jerzy Kukuczka Academy of Physical Education, 40-065 Katowice, Poland; a.zwierzchowska@awf.katowice.pl (A.Z.); d.celebanska@awf.katowice.pl (D.C.); 2 Department of Physiological and Medical Sciences, The Jerzy Kukuczka Academy of Physical Education, 40-065 Katowice, Poland; m.sikora@awf.katowice.pl

**Keywords:** forced sedentariness, sports activity, people with disabilities, anthropometric characteristics

## Abstract

**Simple Summary:**

People with disabilities, especially those with musculoskeletal disabilities, are prone to leading forced sedentary lifestyles due to their limitations. Inactivity or reduced physical activity affect their body composition and physique. There are also athletes among people with disabilities. The sports they practice can compensate for physical inactivity in everyday life. The aim of our study was to demonstrate that forced sedentariness and varied sports activity are factors differentiating between anthropometric characteristics, indices, and body composition of individuals with disabilities, including Polish Para athletes (track and field athletes, sitting volleyball players, and wheelchair rugby players). It was found that non-athletes had the highest levels of obesity indices compared to Para athletes. Furthermore, in the group of Polish Para athletes, sitting volleyball players had the highest values of obesity indices. The results of the present study indicate that forced sedentariness and sports activity among individuals with disabilities differentiate body structure and physique.

**Abstract:**

**Introduction:** Although the assessment of physique and body composition poses methodological, technical, and interpretative difficulties, it is of great importance for the health of people with disabilities. The aim of the study was to demonstrate that sedentariness and sports activity are factors differentiating anthropometric characteristics, indices, and body composition in people with physical disabilities. **Materials and methods**: Fifty-eight people were examined: 48 elite Polish Para athletes, including Paralympic track and field athletes (PTF, *n* = 8), sitting volleyball players (SV, *n* = 15), wheelchair rugby players (WR, *n* = 25), and individuals with cervical spinal cord injury (CSCI, *n* = 10). Body mass (BM), body height (BH), body length (BL), waist circumference (WC), hip circumference (HC), body fat percentage (%FT), and visceral fat rating (VFR) were measured. Furthermore, BMI, BMIcorrected, and body adiposity index (BAI) were evaluated. **Results**: The highest WC, BAI, %FT, and VFR were found for the CSCI group. The type of sport significantly differentiated between anthropometric features, indices, and body composition of the athletes. Sitting volleyball players achieved the highest mean BM (83.9), WC (92.9), HC (103.7), BMI (24.5), BAI (23.4), and VFR (12.6). The highest %FT (28.9) was found in wheelchair rugby players. **Conclusions**: The results of the present study indicate that forced sedentariness and sports activity among individuals with disabilities differentiate body structure and physique.

## 1. Introduction

Nowadays, sedentariness has become part of the lifestyle of modern societies. Decreasing levels of physical activity have been observed across populations worldwide while affecting physique and body composition (body fat distribution). 

Sedentary lifestyles also affect people with disabilities. This is especially true for people with physical and motor disabilities (spinal cord injuries (SCI), amputations, and other etiologies) leading to reduced mobility and, consequently, sedentary lifestyles. Physical inactivity accelerates the process of developing systemic diseases including metabolic ones [1,2]. It has been shown that SCI individuals experience a decline in muscle mass in the limbs and body parts affected by the pathology and an increase in body fat, which can lead to overweight or obesity [3]. Furthermore, SCI patients have lower lean body mass and higher body fat mass compared to the general population [2,4]. Therefore, monitoring of basic anthropometric characteristics is an important element of health care for people with disabilities who additionally experience compensatory internal mechanisms resulting from disability [5].

Para athletes are a specific group in the population of people with disabilities. The sports they practice can compensate for physical inactivity in everyday life, be an antidote for disturbed body homeostasis, and at the same time be used in the prevention of metabolic diseases [6]. Research in this field appears to be particularly difficult because Para athletes are a heterogeneous group. They are characterized by varied physical health status and comorbidities, making it difficult to identify a representative study group [7]. This variation can be observed within a single sport (paravolley rules allow people with both severe and minimal permanent motor disabilities to play). A similar situation, although in a slightly different dimension, occurs in people with SCIs, with athletes with different levels of limitations in movement, strength, and control of the arms, trunk, and legs being assigned to one of seven sport classes, ranging from 0.5 to 3.5 points, i.e., from the most limited (0.5) to the highest (3.5) level of functional ability (according to the classification system adopted by the International Wheelchair Rugby Federation) [8].

An additional difficulty is the need to use specific and varied measurement methods adapted to the needs of amputees or people with lower limb paralysis. Previous studies of body composition of Para athletes have used methods such as air-displacement plethysmography, skinfold measurements [9], BOD POD [10], dual X-ray absorptiometry (DXA) [4,11], and magnetic resonance [3]. These methods are expensive and unavailable to widespread use (e.g., DXA, BOD POD) or require precision and experience of the researcher (skinfold measurement). Therefore, there is an ongoing search for alternative easy-to-use methods for estimating body structure and composition of people with SCI and amputations, which allows physiotherapists, coaches, and athletes to receive information about the physical potential and physique of the Para athlete throughout the training process.

The BMI index commonly used in the general population has some limitations, such as difficult measurement of body weight in wheelchair users who cannot assume the habitual posture and overestimation in amputees. Laughton et al. [12] state that in patients with SCI, a BMI of >22 kg/m^2^ should be taken as indicating the risk of obesity and related chronic diseases. Furthermore, for amputees, calculating the correct BMI requires the use of accurate length parameters concerning individual body segments [13]. A standard BMI formula cannot, therefore, be used and can only be estimated for individuals with limb deficiencies using the Brown-Fisher index by calculating corrected BMI (BMIcor) [14].

Bearing this in mind, the proposal of body adiposity index (BAI) according to Bergman et al. [15] is an alternative method to estimate fatness for individuals who are unable to maintain habitual posture (after paralysis) or those with limb deficiencies. The proposed BAI index, which takes into account body height and hip circumference, has been repeatedly validated across different populations and groups [2,16,17,18]. The sensitivity and efficacy of this index in a group of wheelchair rugby players was confirmed by Zwierzchowska et al. [19].

Although the assessment of body composition and physique of people with disabilities presents many difficulties, both methodological, technical, and interpretative, especially when it concerns amputees, the exploration of this issue is important for the practice of sport, as discussed in studies [5,9,10,11,20,21,22,23].

The aim of the study was to demonstrate that sedentariness and sports activity are factors differentiating anthropometric characteristics, indices, and body composition in people with physical disabilities. It was assumed that the sport practiced influences the parameters of physique and body composition of disabled athletes.

## 2. Materials and Methods

### 2.1. Participants

Fifty-eight men were examined: 48 elite Polish Para athletes, including Paralympic track and field athletes (PTF) (*n* = 8), sitting volleyball players (SV) (*n* = 15), wheelchair rugby players (WR) (*n* = 25), and 10 individuals with cervical spinal cord injury (CSCI). The characteristics of the study group are shown in Table 1. Age and time from injury did not significantly differentiate the participants.

### 2.2. Study Design

The direct observation method was used in the study. Anthropometric characteristics such as body weight (BM), body height (BH), waist circumference (WC), and hip circumference (HC) were measured and used to calculate BMI and BAI indices. Body composition measurements were also performed.

Each time the examinations took place in the morning (Para athletes were tested during training camps before training sessions). Body composition was measured under fasting conditions. Each participant was informed in advance about the procedures and aim of the study. Respondents were allowed to withdraw from the measurements at any stage of the research.

### 2.3. Anthropometric Characteristics

The following anthropometric characteristics were measured: body weight (BM) (using a Charder MS 5410 chair scale), body height (BH) (using a Charder HM-200P stadiometer), and body length (BL) in patients who could not assume a standing position (using a tape measure, in the supine position, from the vertex to the basis points or the end of the longer stump in the case of bilateral amputees). Waist circumference (WC) (using a tape measure, at the midpoint between the lower edge of the last palpable rib and the apex of the iliac crest, at the end of the expiratory phase) and hip circumference (HC) (using a tape measure, placed parallel to the ground taking into account the largest gluteal muscle circumference (WHO STEPS Protocol)) were also measured [24]. In participants who were unable to assume a standing position, HC was measured in the lying position. The above measurements were taken three times, and the final result was expressed as a mean of the three measurements.

### 2.4. Indices

BMI was calculated by using the formula: BMI = weight (kg)/height^2^ (m) [25]. The vast majority of the participants were characterized by limb amputations. Therefore, a standard BMI formula could not be used [2] and BMI could only be estimated. For amputees, the body mass index was calculated twice: using the standard formula (BMI) and taking into account the weight of the amputated body part (BMIcorrected—BMIcor). For Para athletes with limb deficiencies, the calculations were made based on the Brown-Fisher rate [14]. The percentages of total body weight assigned to different body segments were as follows: hand (1%), forearm (2%), arm (3%), head (7%), trunk (43%), thigh (12%), shank (5%), and foot (2%) [14]. The BMI values (BMIcor for amputees) were related to normal values taking into account a cut-off point >25 kg/m^2^ [25]. For wheelchair rugby players, BMI was referenced also taking into account a cut-off point of >22 kg/m^2^ [12,26]. The BAI was calculated using the equation suggested by Bergman et al. [15]: BAI = ((hip circumference [cm])/((height [m])1.5) − 18).

### 2.5. Body Composition

Body fat percentage (%FT) and visceral fat rating (VFR) were evaluated using a Viscan Tanita AB-140 fat analyzer (Tanita Corporation, Tokyo, Japan) using the bioelectrical impedance technique. This is a novel device for direct measurements of body fat of subjects who for various reasons cannot assume an upright posture. The study was conducted according to a standard manufacturer’s protocol (VISCAN). Measurements are taken in under 30 s, which is convenient when working with disabled, critically ill, and elderly patients. Hands are placed on the chest, and the area to be tested is exposed. In subjects with severe spasticity, the lower limbs are stabilized by the person performing the test. Measurements are non-invasive. This is the basis for calculating body composition by an algorithm that includes sex [27,28].

### 2.6. Statistical Analysis

Distributions of the anthropometric characteristics measured (BM, BH, BL, WC, HC), indices (BMI, BMIcor, BAI), and body composition (%FT, VFR) (Kolmogorov–Smirnov test) were verified. Means ($\bar{x}$) and standard deviations (SD) of the variables measured were computed. The correlation of time from injury with variables measured was verified (Spearman correlation). Variation in the variables by sport was verified (analysis of variance with post hoc test for multiple comparisons). The significance level was set at *p* < 0.05.

## 3. Results

### 3.1. Anthropometric Characteristics

**Figure 1:**
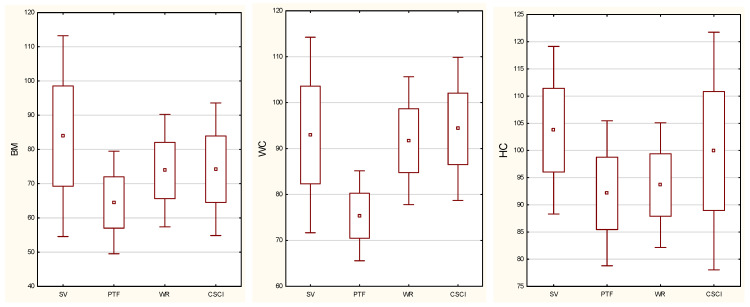
Distribution (mean and SD) of body weight (BM), waist circumference (WC), hip circumference (HC) in the study groups (SV—sitting volleyball players; PTF—Para track and field athletes; WR—wheelchair rugby player; CSCI—cervical spinal cord injury).

The distribution of anthropometric characteristics in the study groups is presented in Figure 1. The highest means of anthropometric characteristics (BM, BH/BL, HC) were found for sitting volleyball players and the highest WC was found for CSCI group. There was variation in the mean values of anthropometric characteristics in the groups studied (BM *p* = 0.0018, WC *p* = 0.0004, HC *p* = 0.0011). Results of the post hoc test are shown in Table 2.

### 3.2. Indices (BMI, BAI)

**Figure 2:**
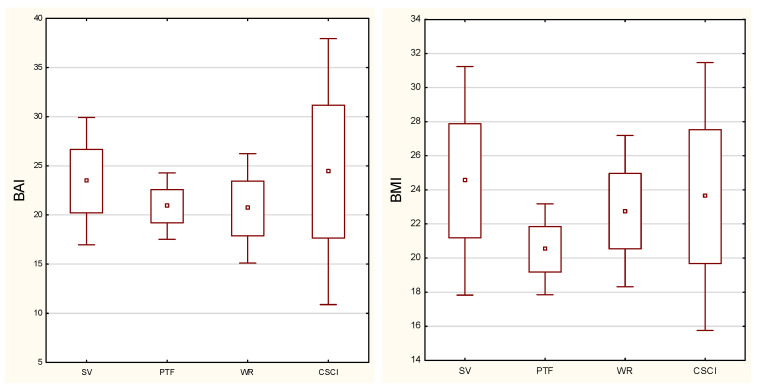
Distribution of body mass index (BMI) and body adiposity index (BAI) in the study groups (SV—sitting volleyball players; PTF—Para track and field athletes; WR—wheelchair rugby player; CSCI—cervical spinal cord injury).

The highest BMI was reported for sitting volleyball and BAI in the CSCI group (Figure 2). The sport practiced was a significant differentiating factor between the groups of athletes for BMI (*p* = 0.0076) and BMIcor, with detailed results of the post hoc test presented in Table 3.

### 3.3. Body Composition

**Figure 3:**
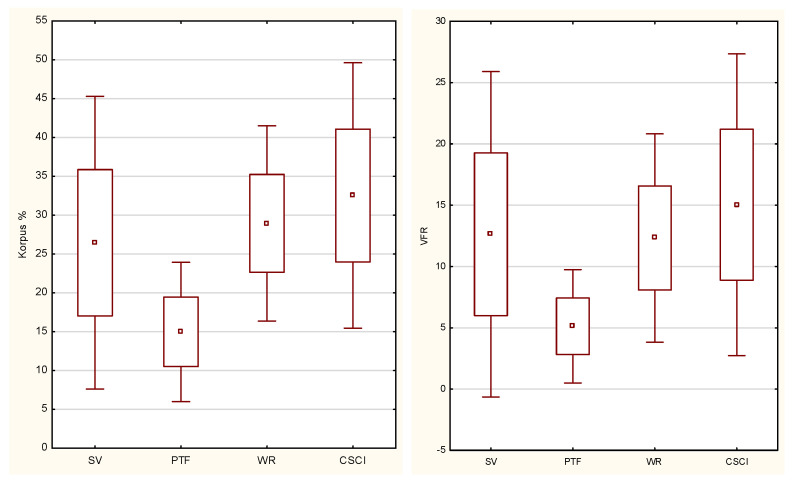
Distribution of body fat percentage (%FT) and viscera fat rating (VFR) in the study groups (SV—sitting volleyball players; PTF—Para track and field athletes; WR—wheelchair rugby player; CSCI—cervical spinal cord injury).

The highest %FT and VFR values were reported in the CSCI group (Figure 3). The sport practiced was a significant differentiating factor between the groups of athletes (%FT *p* = 0.0008, VFR *p* = 0.0009) with detailed results of the post hoc test presented in Table 4.

## 4. Discussion

The aim of this study was to demonstrate that forced sedentary lifestyles and varied sports activities are factors that differentiate between anthropometric characteristics, indices, and body composition of people with physical disabilities. The study included non-athletes and individuals undertaking varied sports (sitting volleyball, wheelchair rugby, Para track and field). It was confirmed that disabled people with sedentary lifestyles (CSCI group) were characterized by the highest values of obesity indices compared to athletes. Among athletes, the highest indices were found in sitting volleyball players.

Studies have repeatedly shown that sedentariness is a factor influencing parameters of body composition and physique [29]. People with motor disabilities are particularly predisposed to sedentary lifestyles. Consequently, they are prone to increased body fat as demonstrated in our study.

A certain percentage of persons with motor disabilities are those who engage in the sports activity. This trend seems to be an antidote to sedentariness by affecting parameters of physique and body composition, which was also confirmed in our study. It was shown that the athletes had lower levels of both body fat and VFR compared to CSCI non-athletes. This is confirmed by the findings of Cavedon et al. [30] who compared the body composition of three groups: wheelchair athletes, non-athletic participants with a physical impairment, and non-athletic able-bodied participants. Wheelchair athletes had a significantly lower body fat mass and % body fat in the whole body and trunk region compared to non-athletic participants with a physical impairment. A study by Willems et al. [22] carried out on two groups of wheelchair players (walkers and non-walkers) indicated no variation in fat mass between groups while at the same time there was significantly lower body mass and lean mass in the walker group.

It was also shown that not only the undertaking of a sports activity but also the type of sports activity can differentiate the anthropometric characteristics, indices, and body composition of disabled athletes. Flueck [11], who studied different groups of athletes with musculoskeletal injuries using wheelchairs, observed a lack of significant variation in body fat and fat-free mass between paraplegics, tetraplegics, and those without spinal cord injury. Flueck [11] used the gold standard method of the evaluation of body composition, i.e., dual-energy X-ray absorptiometry (DXA), while at the same time there was a significant diversification in the participants according to the sport practiced (curling, paracycling (only handcycling athletes), wheelchair rugby, wheelchair basketball, wheelchair racing, table tennis, tennis, badminton, ski alpine, archery, and shooting), which is also consistent with our results. The examinations conducted by Gorla et al. [6], indicated that rugby training (performed four times a week) that incorporates aerobic and anaerobic exercise is an effective means of improving body composition characteristics and indices (decrease in fat mass and increase in lean mass). This is consistent with our findings as wheelchair rugby players are the most sedentary and functionally different group of Para athletes compared to sitting volleyball players, but at the same time, their results were similar. Therefore, the type of sport practiced (not only the type of exercise) is important in stimulating adaptive mechanisms that modify body structure, as the type of disability and functional potential of volleyball players are significantly higher than that of wheelchair rugby players.

Romanov et al. [31] demonstrated a significant association of anthropometric characteristics with athletic performance, thus confirming the importance of physique in sport. With the professionalization of Paralympic sports, it also seems that in this group this factor should be considered important in the selection for sports. In contrast, much of the research on Para athletes concerning the evaluation of anthropometric characteristics and body composition is focused on the characterization of these variables [32], their comparison to fitness test scores [10,33,34], or the search for an accurate and reliable method to estimate body composition in this group [22,23,35]. Few studies have addressed the problems of comparing the characteristics between athletes of different sports [11,22]. Our study confirms the validity of such comparisons, as significant somatic differentiation between sports has been demonstrated, which at the same time provides a reason to identify compensatory mechanisms in body composition as a result of adaptations. 

Therefore, it seems that physiotherapists, coaches, and Para athletes should control and be able to interpret basic anthropometric characteristics and indices. This has implications for both the health of the athletes and the evaluation of their athletic potential.

The limitation of the study is the unequal groups of participants and the high variability of participants within a single sport, as expressed by high standard deviations for time since injury and age, especially in the Para track and field group. However, it should be noted that athletes with disabilities are a specific group. Furthermore, to the best of our knowledge, few studies have compared anthropometric characteristics, indices, and body composition between athletes of different Paralympic sports.

## 5. Conclusions

The results of the present study indicate that forced sedentariness and sports activity among individuals with disabilities differentiate body structure and physique. It seems interesting to identify (apart from the functional potential of a person with a disability) the athletes’ somatic predispositions for the type of sport practiced, which we indicate as a direction for further scientific exploration.

## Figures and Tables

**Table 1:** Characteristics of the group.

Group	Mean Age $\bar{x}$ (sd) [Year]	Mean Time from Injury $\bar{x}$ (sd) [Year]
CSCI (*n* = 10)	32.8 (4.5)	11 (5.5)
PTF (*n* = 8)	36.6 (10.6)	15.3 (14.5)
SV (*n* = 15)	32.8 (8.4)	19.2 (12.1)
WR (*n* = 25)	32.3 (5.2)	12.3 (5.1)

CSCI—cervical spinal cord injury; PTF—Para track and field; SV—sitting volleyball; WR—wheelchair rugby.

**Table 2:** Post hoc test for multiple comparisons of anthropometric characteristics.

	CSCI	Sitting Volleyball Players	Para Track and Field Athletes	Rugby Players
BM				
CSCI	-	0.37	0.32	1.0
Sitting volleyball players	0.37	-	**0.0008**	0.17
Para track and field athletes	0.32	**0.0008**	-	0.15
Rugby players	1.0	0.17	0.15	-
BH				
CSCI	-	0.32	1.0	1.0
Sitting volleyball players	0.32	-	0.34	0.38
Para track and field athletes	1.0	0.34	-	1.0
Rugby players	1.0	0.38	1.0	-
WC				
CSCI	-	1.0	0.001	1.0
Sitting volleyball players	1.0	-	**0.0007**	1.0
Para track and field athletes	**0.001**	**0.0007**	-	**0.001**
Rugby players	1.0	1.0	**0.001**	-
HC				
CSCI	-	0.7	0.9	0.8
Sitting volleyball players	0.7	-		**0.001**
Para track and field athletes	0.9	0.02	-	1.0
Rugby players	0.8	**0.001**	1.0	-

BM—body weight; BH/BL—body height, body length; WC—waist circumference; HC—hip circumference; CSCI—cervical spinal cord injury.

**Table 3:** Post hoc test for multiple comparisons of indices.

	CSCI	Sitting Volleyball Players	Para Track and Field Athletes	Rugby Players
BMI				
CSCI	-	1.0	0.2	1.0
Sitting volleyball players	1.0	-	**0.004**	0.4
Para track and field athletes	0.2	**0.004**	-	0.15
Rugby players	1.0	0.4	0.15	-
BMIcor				
CSCI	-	0.1	0.3	1.0
Sitting volleyball players	0.1	-	**0.00008**	**0.005**
Para track and field athletes	0.3	**0.00008**	-	0.3
Rugby players	1.0	**0.005**	0.3	-
BAI				
CSCI	-	1.0	1.0	0.9
Sitting volleyball players	1.0	-	0.5	0.08
Para track and field athletes	1.0	0.5	-	1.0
Rugby players	0.9	0.08	1.0	-

CSCI—cervical spinal cord injury; BMI—body mass index; BAI—body adiposity index.

**Table 4:** Post hoc test for multiple comparisons of body composition.

	CSCI	Sitting Volleyball Players	Para Track and Field Athletes	Rugby Players
%FT				
CSCI	-	0.37	0.32	1.0
Sitting volleyball players	0.37	-	**0.0008**	0.17
Para track and field athletes	0.32	**0.0008**	-	0.15
Rugby players	1.0	0.17	0.15	-
VFR				
CSCI	-	0.32	1.0	1.0
Sitting volleyball players	0.32	-	0.34	0.38
Para track and field athletes	1.0	0.34	-	1.0
Rugby players	1.0	0.38	1.0	-

CSCI—cervical spinal cord injury; %FT—body fat percentage; VFR—visceral fat rating.

## Data Availability

The data presented in this study are available on request from the corresponding author.
